# L−shaped association of triglyceride glucose index and sensorineural hearing loss: results from a cross-sectional study and Mendelian randomization analysis

**DOI:** 10.3389/fendo.2024.1339731

**Published:** 2024-02-23

**Authors:** Yixuan Wang, Hui Liu, Xinlin Nie, Na Lu, Sheng Yan, Xin Wang, Yuxiang Zhao

**Affiliations:** ^1^ Department of Otolaryngology Head and Neck Surgery, Shaanxi Provincial People’s Hospital, Xi’an, China; ^2^ Yan’an University, Yan’an, China; ^3^ Department of Orthopedic Center, the First Hospital of Jilin University, Changchun, China

**Keywords:** triglyceride-glucose index, fasting blood glucose, sensorineural hearing loss, NHANES, Mendelian randomization

## Abstract

**Background:**

The association between the sensorineural hearing loss (SNHL) and triglyceride-glucose (TyG) index remains inadequately understood. This investigation seeks to elucidate the connection between the TyG index and SNHL.

**Methods:**

In this cross-sectional study, we utilized datasets sourced from the National Health and Nutrition Examination Survey (NHANES). A comprehensive analysis was conducted on 1,851 participants aged 20 to 69, utilizing complete audiometry data from the NHANES database spanning from 2007 to 2018. All enrolled participants had accessible hearing data, and the average thresholds were measured and calculated as both low-frequency pure-tone average and high-frequency pure-tone average. Sensorineural hearing loss (SNHL) was defined as an average pure tone of 20 dB or higher in at least one better ear. Our analysis involved the application of multivariate linear regression models to examine the linear relationship between the TyG index and SNHL. To delineate any non-linear associations, we utilized fitted smoothing curves and conducted threshold effect analysis. Furthermore, we conducted a two-sample Mendelian randomization (MR) study, leveraging genetic data from genome-wide association studies (GWAS) on circulating lipids, blood glucose, and SNHL. The primary analytical method for the MR study was the application of the inverse-variance-weighted (IVW) approach.

**Results:**

In our multivariate linear regression analysis, a substantial positive correlation emerged between the TyG index and SNHL [2.10 (1.80-2.44), p < 0.0001]. Furthermore, using a two-segment linear regression model, we found an L-shaped relationship between TyG index, fasting blood glucose and SNHL with an inflection point of 9.07 and 94 mg/dL, respectively. Specifically, TyG index [3.60, (1.42-9.14)] and blood glucose [1.01, (1.00-1.01)] concentration higher than the threshold values was positively associated with SNHL risk. Genetically determined triglyceride levels demonstrated a causal impact on SNHL (OR = 1.092, p = 8.006 × 10^−4^). In addition, blood glucose was found to have a protective effect on SNHL (OR = 0.886, p = 1.012 × 10^−2^).

**Conclusions:**

An L-shaped association was identified among the TyG index, fasting blood glucose, and SNHL in the American population. TyG index of more than 9.07 and blood glucose of more than 94 mg/dL were significantly and positively associated with SNHL risk, respectively.

## 1 Introduction

Auditory function is pivotal in shaping our social interactions, serving as a fundamental component for both the reception and expression of language, thereby underpinning effective communication. Unfortunately, hearing loss has emerged as a highly prevalent sensory impairment globally. The global prevalence of Sensorineural hearing loss (SNHL) was initially documented by the World Health Organization (WHO) in 1985 (1). According to the latest assessment by the WHO, approximately 466 million individuals, constituting 6.1% of the global population, were affected by disabling hearing loss in 2018. Projections indicate that this figure is anticipated to escalate to 630 million by 2030 and surpass 900 million by 2050 (2). SNHL, characterized by impaired perception of sound and compromised nerve impulse conduction due to lesions in the hair cells of the spiral apparatus, the auditory nerve, or various hearing centers, represents a significant challenge (3, 4). Notably, adults afflicted with SNHL often contend with mental disorders, depression, and heightened occupational stress compared to their unaffected counterparts (1). This predicament not only poses a substantial and intricate economic burden on society and public health but also profoundly influences the overall well-being and mental health of those affected. Effectively addressing and mitigating the impact of SNHL remains an ongoing challenge. In recent years, the correlation between hearing impairments and both type 1 (5) and type 2 (6) diabetes is widely acknowledged. A meta-analysis incorporating cohorts of both type 1 and type 2 diabetes patients revealed a consistent association between diabetes and hearing impairment across both diabetes types. Notably, it is essential to recognize that prediabetes constitutes a risk factor for developing type 2 diabetes and often coexists with the same comorbidities associated with type 2 diabetes (7). Insulin resistance (IR) is a pivotal factor in the progression toward type 2 diabetes (T2D) and is prevalent in the majority of prediabetic (preDM) individuals (8).

IR manifests as a diminished sensitivity and responsiveness to insulin’s actions, often preceding the onset of diabetes mellitus by several years (9). Recent studies increasingly highlight the close association between IR and the aging process. Both IR and SNHL are conditions intertwined with aging, featuring complex and multifactorial pathogeneses. However, despite the acknowledged overlap, there exists a dearth of conclusive information regarding the impact of IR on SNHL. The Triglyceride-Glucose (TyG) index, a composite metric derived from fasting triglyceride and blood glucose levels, serves as an evaluative measure for IR (10). In contrast to conventional methods for assessing insulin resistance (IR) like the hyperinsulinemic-euglycemic clamp technique and the homeostasis model assessment for IR, the TyG index distinguishes itself through its notable cost-effectiveness and straightforward acquisition process (11). Hence, our study aims to elucidate the relationship between the TyG index and SNHL among participants in the National Health and Nutrition Examination Survey (NHANES).

To appraise the influence of the TyG index on the prevalence of SNHL, our initial step involved conducting an observational study utilizing data sourced from the United States population as recorded in the NHANES database (12). Moreover, we applied a Mendelian randomization (MR) approach to thoroughly examine the causal association between circulating lipid and glucose levels and the risk of SNHL. This method facilitates the deduction of causal relationships using genetic instrumental variables (IVs). Leveraging the attributes of random assortment and consistency within germline genotypes, MR holds the potential to mitigate biases stemming from confounding and reverse causation, challenges commonly encountered in observational epidemiology (13).

## 2 Method

We utilized genome-wide association studies (GWAS) data from publicly available trials and United States population data extracted from the NHANES database. All the datasets employed in this study have received approval from the ethics committee, obviating the need for separate ethical clearance.

### 2.1 Cross-sectional study

#### 2.1.1 Study population and design

**Figure 1:**
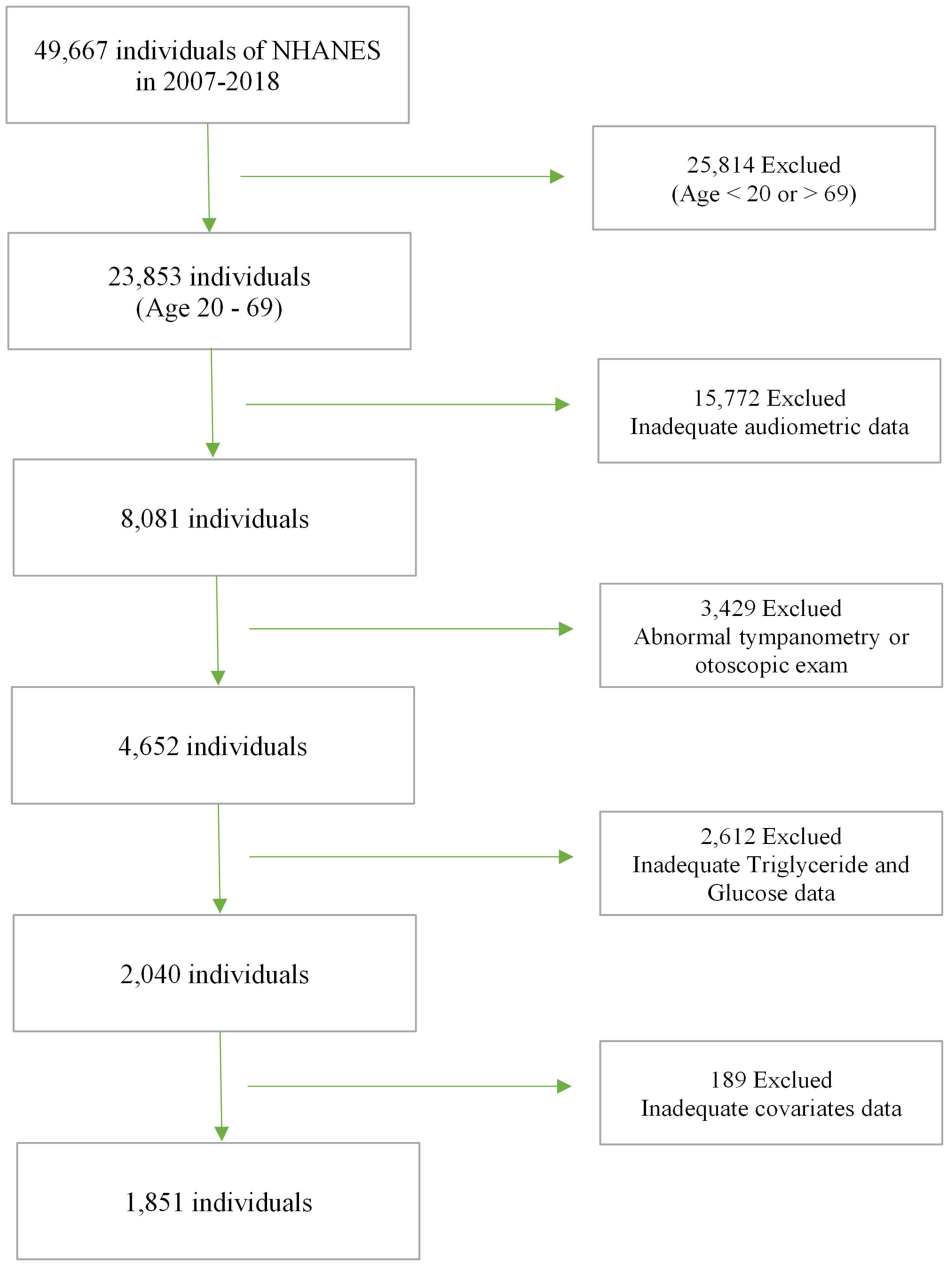
Flow chart of study participants.

The NHANES stands as a pivotal research endeavor, intricately crafted to evaluate the health and nutritional status of both adults and children residing in the United States. Orchestrated by the National Center for Health Statistics (NCHS) under the aegis of the Center for Disease Control and Prevention, the survey protocol underwent meticulous scrutiny and received approval from the National Center for Health Statistics Institutional Review Board. Conducted biennially with an approximate sample size of 5,000, NHANES systematically gathers comprehensive information on family health and nutrition. This protocol adheres to the highest standards of research ethics, safeguarding the rights of participants through the acquisition of informed, written consent from all study individuals. For our investigation into the association between the TyG and SNHL, we selected five NHANES cycles spanning from 2007 to 2012 and 2015 to 2018. These cycles were chosen due to the inclusion of complete variables necessary for calculating triglyceride (TG), blood glucose, and audiometric data, all processed using standardized protocols. Our analysis adhered to stringent exclusion criteria: individuals aged <20 years or >69 years, those with abnormal tympanometry or otoscopic exam results, and those lacking complete data on TG, blood glucose, audiometric information, and covariates. Initially enrolling 49,667 participants, our final analysis incorporated 1,851 eligible individuals after implementing the aforementioned exclusion criteria. (**Figure 1**).

#### 2.1.2 TyG index measurement

The TyG index was calculated employing the formula: Ln [fasting TG (mg/dL) × fasting glucose (mg/dL)/2]. To assess triglyceride (TG) and fasting glucose levels, enzymatic assays were conducted using Roche Modular P and Roche Cobas 6000 chemistry analyzers, respectively. The measurement of fasting glucose utilized the hexokinase-mediated reaction on Roche/Hitachi Cobas C 501 chemistry analyzers.

#### 2.1.3 Definition of sensorineural hearing loss

SNHL was defined as a mean pure-tone hearing threshold exceeding 20 dB, with the exclusion of potential mixed or conductive hearing loss (9, 14). In the NHANES 2007-2018 audiometric examination, the assessment comprised a pre-exam audiometric questionnaire, a concise otoscopic screening involving a physical examination of the ear canals and eardrums, tympanometry, and pure-tone air conduction audiometry. For a diagnosis of SNHL, specific criteria were adhered to: a mean pure-tone hearing threshold exceeding 20 decibels, the absence of symptoms such as recent colds within the past 24 hours, normal otoscopic findings, and a tympanogram indicating type A with a peak conductance of at least 0.3 milliliters. In accordance with established protocols for assessing hearing in this age group, the low-frequency pure-tone average was computed by taking the average of air conduction pure-tone thresholds at 0.5, 1, and 2 kHz. Similarly, the high-frequency pure-tone average was determined by averaging air conduction pure-tone thresholds at 3, 4, 6, and 8 kHz (15).

#### 2.1.4 Audiometric measures

The NHANES protocol for audiometric evaluations included a comprehensive approach with a hearing questionnaire, otoscopic examination, tympanometry, and pure-tone air conduction audiometry. During the audiometric sessions, air-conduction thresholds were meticulously assessed for each ear at frequencies of 0.5, 1, 2, 3, 4, 6, and 8 kHz, spanning an intensity range from −10 to 120 decibels. Specifically, testing at 1 kHz involved duplicate measurements of the threshold for each ear across an intensity spectrum of −10 to 120 decibels. In instances where the disparity between the two tests exceeded 10 decibels, the results were deemed unacceptable. Subsequently, the outcomes from the initial 1,000 Hz test were exclusively utilized in our analytical framework.

#### 2.1.5 covariates

Demographic and health-related data were obtained through NHANES household interviews, capturing details such as age, gender, race/ethnicity, educational attainment, and family income-to-poverty ratio. Laboratory test results, including serum cotinine, were also collected. Our analysis focused on individuals aged 20 to 69 years, within the NHANES hearing exam’s target age range. Race classifications encompassed Mexican American, Other Hispanic, Non-Hispanic White, Non-Hispanic Black, and Other Race. Educational attainment was delineated into categories: Less than 9th grade, 9th-11th grade, High school graduate/GED or equivalent, Some college or AA degree, and College graduate or above.

In our quantitative exposure assessment study, we opted to utilize plasma cotinine as a marker of active smoking due to its longer half-life in the blood compared to nicotine. Body mass index (BMI) was calculated by dividing weight in kilograms by the square of height in meters. Additionally, clinical markers such as low-density lipoprotein cholesterol (LDL-C), high-density lipoprotein cholesterol (HDL-C), total cholesterol (TC) were collected from NHANES laboratory data. To enhance the transparency of our methodology, we refer to previous research for detailed information on the variables (16, 17). Comprehensive measurements of all study variables are available at www.cdc.gov/nchs/nhanes/.

#### 2.1.6 Statistical analysis

The statistical analyses were conducted using R (version 4.3.0) and Empower Stats (version 2.0). Demographic characteristics of the subjects were compared across SNHL status groups using a t-test and chi-square test. We calculated beta values and 95% confidence intervals using multivariate logistic regression analysis between TyG index and SNHL.

Model 1 was unadjusted, Model 2 incorporated adjustments for age, race, and gender, while Model 3 included additional adjustments for age, gender, race, education level, family income-to-poverty ratio, LDL-C, HDL-C, TC, Cotinine, and BMI. Simultaneous smoothed curve fits were performed after adjusting these variables.

Furthermore, a threshold effects analysis model was employed to scrutinize the relationship and identify the inflection point between TyG index, fasting blood glucose, and SNHL. Subgroup analyses and interaction tests were undertaken to investigate the relationship between the TyG index and SNHL. The threshold for statistical significance was defined as p < 0.05.

### 2.2 Mendelian randomization study

#### 2.2.1 Study design and data sources

We filter independent single-nucleotide polymorphisms (SNPs) to constitute instrumental variables (IVs) associated with circulating lipids, blood glucose and performed a two-sample Mendelian randomization analysis with SNHL GWAS data, respectively.

GWAS statistics for SNHL were from FinnGen consortium (18). The data included 364,223 Finnish adult subjects (32,487 cases and 331,736 controls). SNHL is characterized by hearing loss originating from the inner ear or sensory organ (including the cochlea and associated structures) or the vestibulocochlear nerve (cranial nerve VIII). Circulating lipids, blood glucose related GWAS from the UK Biobank (UKB) database, all subjects included in the analysis were from European ancestry.

#### 2.2.2 IVs Selection

Screening of IVs according to the following criteria: 1. Screening of strongly correlated SNPs by p < 5 × 10^-8^; 2. Removal of linkage disequilibrium according to clumping r² = 0.001 and kb = 10,000. Subsequent exclusion of SNHL-associated SNPs. The instrumental SNPs are shown in the **Supplementary Table S1-5**.

#### 2.2.3 Statistical analysis

The inverse-variance weighted (IVW) method (19) was used as its principal analytical method. Simultaneously, the MR-Egger and weighted median methods were employed to enhance the precision of estimates. These approaches were implemented to address variant heterogeneity and mitigate the potential influence of pleiotropy.

The “leave-one-out analyses”, the funnel plot and MR-Egger intercept test were used to assess pleiotropy. Cochran’s Q test was used to detect heterogeneity. To enhance control over the potential influence of pleiotropy, we employed the MR-PRESSO method to conduct a thorough assessment of heterogeneity and identify instances of horizontal pleiotropy.

The R software (Version 4.3.0) was used to data analysis, the p-value less than 0.05 is regarded as a strong correlation.

## 3 Results

### 3.1 Cross-sectional study

#### 3.1.1 Baseline characteristics

A total of 1,851 participants were included in the study, comprising 48.14% males and 51.86% females, with an average age of 42.47 ± 13.99 years. Among the participants, 835 (45.11%) exhibited SNHL. Significant differences in age, gender, race, education level, Cotinine, LDL-C, HDL-C, TC, BMI, and TyG index were observed between individuals with and without SNHL (all p<0.05). SNHL patients, compared to those without, were more likely to be male, Non-Hispanic White, have a lower education level, be smokers, exhibit advanced age, higher BMI, elevated LDL, TC, and TyG index levels, and reduced HDL-C levels in our study (all p < 0.05). **Table 1** presents the clinical and biochemical characteristics of the subjects based on whether they were SNHL patients.

**Table 1:** Clinical characteristics of all 1,851 subjects among subjects with SNHL and without SNHL.

Characteristics	Control	SNHL	*P*-value
	n=1016	n=835	
Age, years	35.49 ± 10.97	51.24 ± 11.97	<0.0001
Gender (%)			<0.0001
Male	43.03	55.03	
Female	56.97	44.97	
Race (%)			<0.0001
Mexican American	9.17	7.05	
Other Hispanic	7.08	6.30	
Non-Hispanic White	63.69	73.51	
Non-Hispanic Black	11.34	6.74	
Other Race	8.71	6.39	
Education level (%)			<0.0001
<9th grade	2.92	4.94	
9–11th grade	7.18	9.60	
High school grade/GED or equivalent	16.11	24.74	
Some college or AA degree	34.69	27.12	
College graduate or above	39.09	33.60	
PIR, mean	2.93 ± 1.65	3.07 ± 1.63	0.0570
Cotinine, ng/ml	45.81 ± 110.09	68.78 ± 142.54	<0.0001
LDL-cholesterol, mmol/L	111.67 ± 32.92	120.22 ± 35.18	<0.0001
HDL-cholesterol, mmol/L	55.52 ± 15.67	53.78 ± 19.02	0.0305
TC, mmol/L, mean	187.95 ± 38.56	199.99 ± 39.44	<0.0001
BMI, kg/m2	28.37 ± 6.85	29.93 ± 6.74	<0.0001
TyG index	8.38 ± 0.60	8.70 ± 0.65	<0.0001

Mean ± SD for continuous variables: the P value was calculated by weighted linear regression model.

PIR, the ratio of family income to poverty; TC, Total Cholesterol; BMI, body mass index; TyG, triglyceride-glucose; SNHL, sensorineural hearing loss.

#### 3.1.2 The association between TyG index and SNHL

Our results unveiled a substantial association between an elevated TyG index and SNHL. In both our unadjusted model (OR=2.10; 95% CI, 1.80–2.44, p < 0.0001) and the minimally adjusted model (OR=1.29; 95% CI, 1.07–1.56, p < 0.01), each incremental unit increase in the TyG index correlated with a 29% heightened prevalence of SNHL. To bolster the robustness of our findings, a sensitivity analysis was conducted, categorizing the TyG index into tertiles. Individuals in the highest TyG index tertile manifested a noteworthy 160% elevated risk of SNHL compared to their counterparts in the lowest tertile, demonstrating statistical significance (OR=2.60; 95% CI, 2.07–3.28, p < 0.0001). Moreover, participants in the middle TyG index tertile also exhibited a heightened risk of SNHL compared to the lowest tertile, reaching statistical significance (OR=1.37; 95% CI, 1.09–1.73, p = 0.0072) (**Table 2**).

**Table 2:** The associations between TyG index and SNHL.

Exposure	Model I OR (95% CI) *P*	Model II OR (95% CI) *P*	Model III OR (95% CI) *P*
TyG index	2.10 (1.80, 2.44) <0.0001	1.29 (1.07, 1.56) 0.0084	1.20 (0.75, 1.91) 0.4510
TyG index classification			
Low TyG index (<8.23)	Reference	Reference	Reference
Middle TyG index (8.23-8.78)	1.37 (1.09, 1.73) 0.0072	0.91 (0.68, 1.22) 0.5314	0.76 (0.54, 1.06) 0.1047
High TyG index (>8.78)	2.60 (2.07, 3.28) <0.0001	1.29 (0.96, 1.73) 0.0860	0.88 (0.53, 1.47) 0.6307
*P* for trend	2.16 (1.80, 2.61) <0.0001	1.24 (0.98, 1.57) 0.0706	0.86 (0.57, 1.30) 0.4764

Model I: None covariates were adjusted;

Model II: Gender, age and race were adjusted;

Model III: Gender, age, race, education level, PIR, LDL-cholesterol, HDL-cholesterol, TC, Cotinine and BMI were adjusted.

PIR, the ratio of family income to poverty; TC, Total Cholesterol; BMI, body mass index; TyG, triglyceride-glucose; SNHL, sensorineural hearing loss.

#### 3.1.3 Subgroup analyses

We discovered erratic correlations between the TyG index and SNHL in subgroup analyses stratified by BMI. As shown in **Table 3**, there was no significant correlation in the results of the interaction test for any of the three subgroups of BMI (P for interaction>0.05), which indicates that there is no evidence that this unfavorable correlation between TyG index and SNHL is related to BMI.

**Table 3:** Subgroup analysis for the association between TyG index and SNHL.

Subgroup	OR(95%CI)	*P* for interaction
BMI		0.971
<25	1.25 (0.72, 2.17) 0.4366	
25-30	1.22 (0.65, 2.29) 0.5388	
≥30	1.30 (0.75, 2.25) 0.3476	

Gender, age, race, education level, PIR, LDL-cholesterol, HDL-cholesterol, TC and Cotinine were adjusted. In the subgroup analyses, the model is not adjusted for the stratification variable itself.

PIR, the ratio of family income to poverty; TC, Total Cholesterol; BMI, body mass index; TyG, triglyceride-glucose; SNHL, sensorineural hearing loss.

#### 3.1.4 The detection of nonlinear relationships

**Figure 2:**
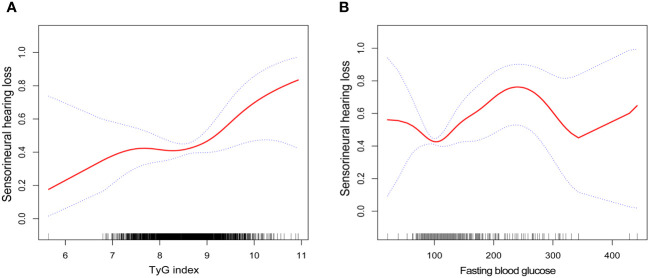
Smooth curve fittings. **(A)** Association between TyG index and SNHL, Gender, age, race, education level, BMI, PIR, LDL-cholesterol, HDL-cholesterol, TC and Cotinine were adjusted; **(B)** Association between fasting blood glucose and SNHL, Gender, age, race, education level, BMI, PIR, TG, LDL-cholesterol, HDL-cholesterol, TC and Cotinine were adjusted. The solid and dotted lines represent the estimated values and their corresponding 95% CIs, respectively.

By smoothing curve fitting (penalized spline method), we found an L-shaped relationship between the TyG index and SNHL (**Figure 2**). We then carried out a further threshold effect analysis of the non-linear relationship (**Table 4**). Our analysis revealed an inflection point for the TyG index at 9.07. No association with SNHL was observed when the TyG index was less than 9.07 (HR 1.09, 95% CI 0.68, 1.77). However, a baseline TyG index exceeding 9.07 exhibited a significant and positive association with SNHL risk (HR 3.60, 95% CI 1.42-9.14). Utilizing similar statistical methods, we investigated the relationship between fasting blood glucose, TG, and SNHL. We identified an L-shaped relationship between fasting blood glucose and SNHL as well (**Figure 2**), with an inflection point at 94 mg/dL. This implies a substantial positive correlation with the risk of SNHL when the blood glucose concentration surpassed 94 mg/dL (HR 1.01, 95% CI 1.00-1.01) (**Table 5**).

**Table 4:** Threshold effect analysis of TyG index and SNHL.

	Adjusted HR (95% CI), *P*-value
Fitting by the standard linear model	1.20 (0.75, 1.91) 0.4510
Fitting by the two-piecewise linear model	
Inflection point	9.07
TyG index < 9.07	1.09 (0.68, 1.77) 0.7138
TyG index ≥ 9.07	3.60 (1.42, 9.14) 0.0070
*P* for Log-likelihood ratio	0.004

Gender, age, race, education level, BMI, PIR, LDL-cholesterol, HDL-cholesterol, TC and Cotinine were adjusted.

PIR, the ratio of family income to poverty; TC, Total Cholesterol; BMI, body mass index; TyG, triglyceride-glucose; SNHL, sensorineural hearing loss.

**Table 5:** Threshold effect analysis of fasting blood glucose and SNHL.

	Adjusted HR (95% CI), *P*-value
Fitting by the standard linear model	1.00 (1.00, 1.01) 0.0300
Fitting by the two-piecewise linear model	
Inflection point	94mg/dL
Fasting Glucose < 94 mg/dL	0.98 (0.95, 1.00) 0.0948
Fasting Glucose ≥ 94 mg/dL	1.01 (1.00, 1.01) 0.0091
*P* for Log-likelihood ratio	0.040

Gender, age, race, education level, BMI, PIR, TG, LDL-cholesterol, HDL-cholesterol, TC and Cotinine were adjusted.

PIR, the ratio of family income to poverty; TG, triglyceride; TC, Total Cholesterol; BMI, body mass index; SNHL, sensorineural hearing loss.

### 3.2 Mendelian randomization study

#### 3.2.1 Selection of instrumental variables

A total of 362, 177, 196, and 313 index SNPs were chosen for the genetic prediction of HDL-C, LDL-C, TC, and TG, respectively. In the case of blood glucose prediction, 130 index SNPs were employed. The F statistics for these genetic instruments surpassed the threshold of 10, signifying their robustness as strong instruments.

#### 3.2.2 Causal effect estimates

**Figure 3:**
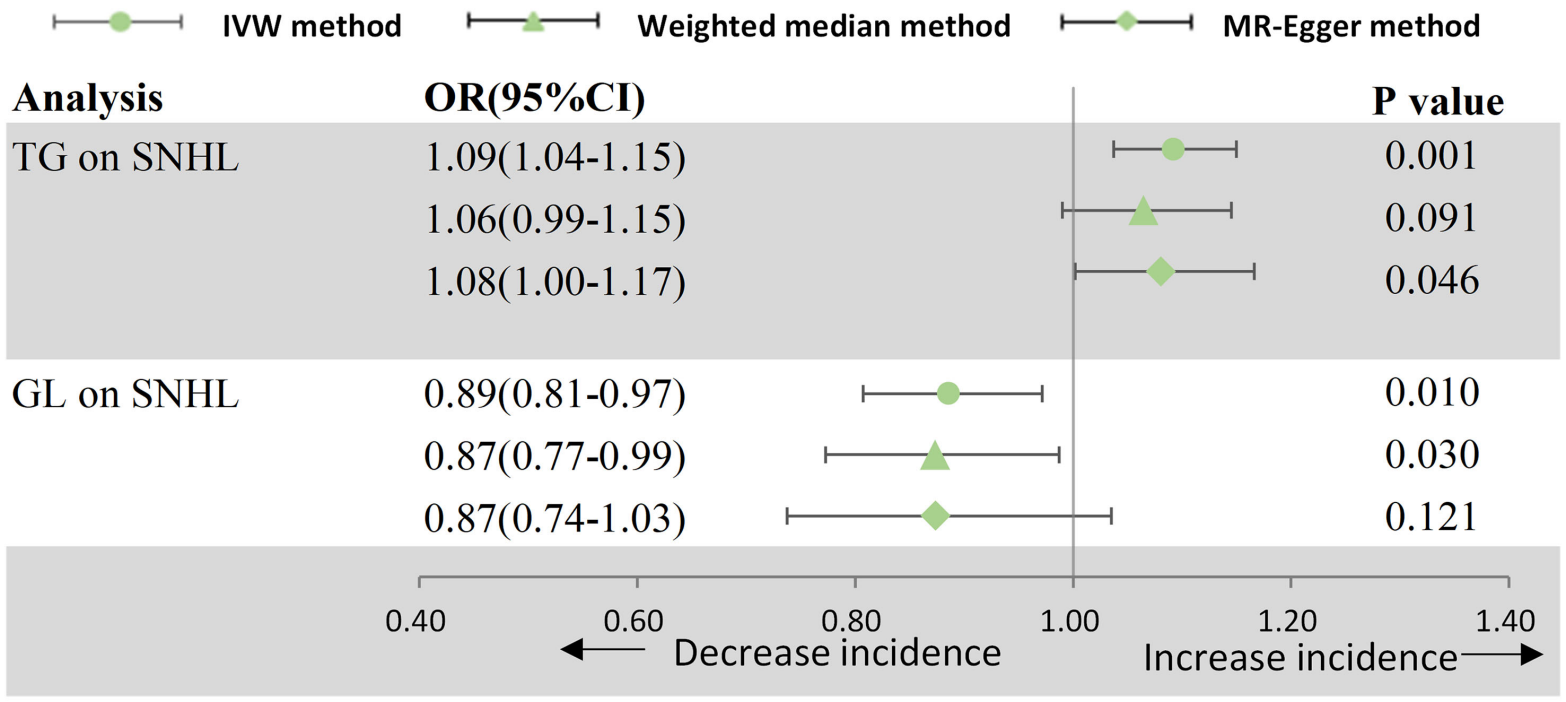
Forest plot for MR analyses. OR, odds ratio; CI, confidence interval; IVW, Inverse-variance weighted; SNHL, sensorineural hearing loss; TG, triglyceride; GL, glucose.

The results of estimating the causal impact of circulating lipids and blood glucose on SNHL are showed in **Figure 3**. In the MR study, utilizing genetically predicted circulating lipids, a statistically correlation between TG and an increased risk of SNHL was identified (OR = 1.092, 95% CI: 1.037–1.150, p = 8.006 × 10^−4^). This association remained robust across the MR-Egger methods. Despite Cochran Q-derived p < 0.05, indicating heterogeneity, the utilization of the random-effects IVW method justified this observed heterogeneity (20). The MR-Egger intercept-derived p-value (> 0.05) suggested the absence of pleiotropy. Both the MR-PRESSSO and leave-one-out plot detected no outliers. However, neither high-density lipoprotein cholesterol (HDL-C) (p = 0.105), low-density lipoprotein cholesterol (LDL-C) (p = 0.536), nor total cholesterol (TC) (p = 0.717) demonstrated a causal effect on SNHL. Subsequently, a significant causal effect of blood glucose levels on SNHL was observed, indicating a decreased risk (OR = 0.886, 95% CI: 0.807–0.972, p = 1.012 × 10−2).

**Table 6** provides comprehensive results of Cochran’s Q test and MR-Egger intercept test. Additionally, scatter plots, funnel plots, and “leave-one-out analysis” plots are included in **Supplementary Figure S1, 2**.

**Table 6:** Significant estimates for MR analysis between circulating lipids, blood glucose, and SNHL.

Exposure	Outcome	IVW-derived P value	OR (95% Confidence intervals)	Cochran’s Q derived P value	MR-Egger intercept derived P value
triglyceride	SNHL	**8.006 × 10^−4^**	1.09 (1.04-1.15)	6.58×10^-10^	0.59
blood glucose	SNHL	**1.012 × 10−2**	0.89 (0.81-0.97)	1.74×10^-4^	0.72

MR, mendelian randomization; Inverse-variance weighted; OR, odds ratio; SNHL, sensorineural hearing loss.

The bold values provided implies significant correlation.

## 4 Discussion

In our cross-sectional study involving 1,851 participants, we present, to our knowledge, the first retrospective investigation with a notably substantial sample size examining the association between the TyG index and SNHL patients. Our study identified an L-shaped relationship between the TyG index and SNHL patients in this cohort, revealing a distinctive turning point at 9.07. This indicates a significant association between elevated TyG index levels and an increased risk of SNHL within a specified range. Notably, our examination of the relationship between fasting glucose and SNHL also revealed an L-shaped pattern, with an inflection point at a fasting glucose level of 94 mg/dL. This implies that maintaining glucose levels within a certain range may impart a protective effect on hearing—a nuanced observation seldom discussed in existing literature. By uncovering these intricate relationships, our study offers valuable insights that could inform strategies for reducing the risk of SNHL and, consequently, contribute to shaping clinical and dietary guidelines. Our findings underscore the importance of individuals with insulin resistance taking proactive measures to prevent hearing loss. This includes maintaining blood glucose within a specific range and adopting a balanced diet. Notably, the broader observation that adherence to a healthy diet correlates with a reduced risk of hearing loss has recently been confirmed in the Nurses’ Health Study II. Participants who adhered to a Mediterranean-style diet or a diet low in processed foods and rich in dietary fiber were less likely to experience hearing loss during a 20-year follow-up period (21). Additionally, individuals with diabetes should be cognizant of the potential association between a higher TyG index and SNHL.

The role of lipids in the pathogenesis of SNHL is extensively discussed in the literature. The cochlea, being an end organ, relies on a continuous supply of nutrients and oxygen to maintain its physiological functions. It exhibits heightened sensitivity to fluctuations in blood circulation (22). Elevated blood viscosity may diminish blood supply to the inner ear, potentially causing inner ear damage (23). Disorders of lipid metabolism can lead to lipid deposition in cochlear hair cells and harm to cochlear nerve cells, impeding nerve conduction (24), Hyperlipidemia is frequently observed in individuals with SNHL (24, 25). and studies have shown that dietary control and lipid-lowering therapy can ameliorate hearing loss associated with cholesterolemia (26). Conversely, the relationship between blood glucose and hearing loss has been approached differently in previous research. While some argue that the auditory system requires glucose for its intricate signal processing and high-energy utilization, others propose that hyperglycemia may adversely affect the cochlea. Increased glucose exposure, even for brief periods, can trigger a metabolic cascade disrupting the cochlea anatomically and physiologically (27). Studies in animal models suggest that combining hyperglycemia with hyperlipidemia and atherosclerosis could exacerbate age-related hearing loss in apolipoprotein E (ApoE)-deficient male mice (28). However, there are conflicting findings, with other research demonstrating that glucose supplementation can protect cochlear hair cells against oxidative stress and noise-induced hearing loss (29), aligning with our Mendelian randomization study that indicates serum glucose has a protective effect against neurological hearing loss. In our NHANES cohort study, we identified an L-shaped relationship between fasting blood glucose and SNHL, with the inflection point at 94 mg/dL. This sheds light on potential explanations for divergent conclusions in prior studies: blood glucose levels exceeding 94 mg/dL increase the risk of sensorineural deafness, emphasizing the importance of maintaining blood sugar levels within a specific concentration range for hearing protection. This phenomenon may be due to the fact that the auditory system is dependent on glucose and high energy for complex signal processing. However, when hyperglycaemia persists, it adversely affects auditory system function through mechanisms such as microvascular lesions, advanced glycosylation end-products and excessive reactive oxygen species (ROS) production, ultimately impairing cochlear function. For instance, the vasodilatory effects of nitrous oxide are compromised in the presence of hyperglycemia and its associated advanced glycosylation end-products, triggering protein kinase C activation and diminishing nitric oxide synthase production (30). Maintaining a critical balance of nitric oxide is crucial for the optimal long-term health of cochlear sensory and support cells. Nitric oxide is strategically situated in key cochlear blood vessels, including the spiral modiolar, basilar membrane, and spiral osseous lamina vessels. Additionally, it is present in vessels proximate to the spiral ganglion, inner and outer hair cells (31, 32). Nitric oxide plays a pivotal role in regulating the cochlear vascular endothelium by responding to ATP-induced increases in cochlear blood flow. Furthermore, it contributes to antithrombotic activity, as well as the regulation of vascular tone and cellular growth (33).

Emerging evidence from animal models underscores the pivotal role of mitochondrial dysfunction and oxidative stress in SNHL pathogenesis (34–36). Mitochondria, as cellular organelles, play crucial roles in cellular activities including metabolism, energy production, apoptosis, and redox signaling (37). Thus, mitochondria become important regulators of cell survival and death. Mitochondria produce ROS by oxidative phosphorylation. The ROS molecules, superoxide anion and hydroxyl radicals that make up ROS, as well as hydrogen peroxide, play crucial roles as cell signaling molecules in cell proliferation, gene expression, and survival (37). Under physiological conditions, ROS levels in cells are regulated by various endogenous antioxidant enzymes. This regulation is essential for the maintenance of cellular homeostasis. However, an imbalance between ROS production and the cellular antioxidant defense system leads to oxidative stress. Oxidative stress in turn causes irreversible damage to cellular components such as proteins, DNA, lipids and other macromolecules. This damage impairs physiological functions and may ultimately lead to cell death. Given the cochlea’s high metabolic demands, particularly in hair cells, vascular striae, and spiral ganglion neurons, these structures are susceptible to the detrimental effects of mitochondria ROS (37). ROS have been identified in cochlear tissue immediately following noise exposure (38). These ROS persist for 7–10 days afterward, spreading from the basal end of the organ of Corti to the apical turn. Additionally, the reactive nitrogen species product peroxynitrite, formed by the combination of nitric oxide and superoxide, has been observed in this context (39). The prolonged presence of oxidative stress resulting from these species can lead to delayed and sustained cochlear injury. Free radicals, comprising ROS and reactive nitrogen species, elicit damage by interacting with various cellular components such as DNA, proteins, cytosolic molecules, cell surface receptors, and membrane lipids. Mitochondria-generated ROS induce lipid peroxidation in the cochlea, giving rise to byproducts like malondialdehyde and 4-hydroxynonenal (40). This process overwhelms the cochlear antioxidant enzyme system, including superoxide dismutase, catalase, glutathione peroxidase, and glutathione reductase, ultimately depleting glutathione—the endogenous antioxidant. The consistent delivery of glucose from the bloodstream ensures that the demanding energy requirements of neurons in the brain are met, supporting physiologic brain function through ATP production (41). Impaired glucose metabolism in the brain is implicated in various neurological disorders, including neurological hypoglycemia, epilepsy, Alzheimer’s disease, and Parkinson’s disease (42–45). Short-term glucose supplementation has also been demonstrated as crucial for tolerating acute oxidative stress (46).

The risk of SNHL escalates when blood glucose surpasses the identified inflection point. Elevated blood glucose levels can induce various adverse physiological changes, including mitochondrial disruption and the inhibition of ATP production linked to aberrant oxidative phosphorylation (47). While the association between type 2 diabetes and hearing impairment has been acknowledged for some time, recent research underscores the broader impact of IR on diverse conditions, such as diabetic complications, occurring prior to the onset of diabetes (48–50). Although the mechanisms underpinning insulin resistance are not entirely elucidated, there is emerging evidence implicating oxidative stress and impaired mitochondrial function (51). Building upon this understanding, our hypothesis posits that insulin resistance impedes ATP production by elevating blood glucose levels, concurrently influencing the body’s glucose utilization. This, in turn, results in decreased production of NADPH—a pivotal cofactor in cellular detoxification and the antioxidant system. Consequently, cellular dysfunction ensues, compromising cell viability and culminating in functional decline (52). The diminished protective capacity against oxidative stress due to insulin resistance could heighten the risk of hearing damage, with oxidative stress reciprocally exacerbating insulin resistance. Recent findings supporting the protective role of G6PD overexpression against oxidative stress and age-related hearing loss further lend credence to our speculations (52). In our current study, we sought to explore the relationship between insulin resistance and SNHL, employing the TyG index as a surrogate measure. The TyG index, derived from fasting triglycerides and fasting blood glucose, serves as an evaluative metric for insulin resistance. Over the past decade, interest in the TyG index has surged, with demonstrated superiority over the HOMA-IR model in assessing insulin resistance in both diabetic and non-diabetic populations (53). Our findings in the present study reveal an L-shaped relationship between the TyG index and SNHL within the U.S. population. Notably, a TyG index exceeding 9.07 was significantly and positively associated with SNHL risk, elucidating an unfavorable correlation between insulin resistance and hearing loss.

Our study is subject to certain limitations that warrant consideration. Firstly, its cross-sectional nature precludes the establishment of temporal relationships, introducing a constraint in discerning causality. Despite our efforts to adjust for various pertinent confounding factors, the potential influence of additional confounders remains a challenge, necessitating cautious interpretation of our findings. Furthermore, the diagnosis of SNHL in our study predominantly relied on pure-tone audiometry, complemented by normal otoscopic examination and tympanograms with peaks >0.3 ml. This methodology may introduce bias towards the conductive component in hearing loss. However, amidst these limitations, our study boasts notable strengths. Leveraging a nationally representative sample, our findings are reflective of a diverse and gender-inclusive adult population in the United States. Furthermore, our study employed a substantial sample size, enabling subgroup analyses and enhancing the validity and generalizability of the findings. This large sample size contributed to the depth and granularity of the investigation. Finally, we incorporated Mendelian randomization to validate the reliability of observational studies by inferring causality from a genetic perspective.

## Data availability statement

The original contributions presented in the study are included in the article/**Supplementary Material**. Further inquiries can be directed to the corresponding authors.

## Ethics statement

All procedures conducted by NHANES and GWAS studies involving human participants adhered to the ethical standards of the relevant institutional and/or national research committee. The studies were in accordance with the principles outlined in the 1964 Helsinki Declaration and its subsequent amendments or comparable ethical standards.

## Author contributions

YW: Data curation, Investigation, Methodology, Software, Writing – original draft, Writing – review & editing. HL: Writing – review & editing. XN: Writing – original draft, Writing – review & editing. NL: Investigation, Writing – review & editing. SY: Investigation, Writing – review & editing. XW: Writing – review & editing. YZ: Writing – review & editing.
