# Supplementary material for: L−shaped association of triglyceride glucose index and sensorineural hearing loss: results from a cross-sectional study and Mendelian randomization analysis
Source: Front Endocrinol (Lausanne). 2024 Feb 23;15:1339731. doi: 10.3389/fendo.2024.1339731 (PMC10921358; doi:10.3389/fendo.2024.1339731)
Supplement: Supplementary file 1 [file DataSheet_1.docx]

Supplementary Figure

**L‑shaped association of Triglyceride Glucose Index and Sensorineural hearing loss: results from a cross-sectional study and Mendelian randomization analysis**

Yixuan Wang, MD, Hui Liu, PhD, Xinlin Nie, MD, Na Lu, MD, Sheng Yan, MD, Xin Wang, PhD, Yuxiang Zhao, MD.

**Figure S1.** IVW estimates of significant results from blood glucose on SNHL. (a) Scatter plots from genetically predicted blood glucose on SNHL; (b) Leave-one-out plot from genetically predicted blood glucose on SNHL; (c) Funnel plots from genetically predicted blood glucose on SNHL.

**Figure S2.** IVW estimates of significant results from triglyceride on SNHL. (a) Scatter plots from genetically predicted triglyceride on SNHL; (b) Leave-one-out plot from genetically predicted triglyceride on SNHL; (c) Funnel plots from genetically predicted triglyceride on SNHL.
